# Identification of promising host-induced silencing targets among genes preferentially transcribed in haustoria of *Puccinia*

**DOI:** 10.1186/s12864-015-1791-y

**Published:** 2015-08-05

**Authors:** Chuntao Yin, Samantha I. Downey, Naeh L. Klages-Mundt, Sowmya Ramachandran, Xianming Chen, Les J. Szabo, Michael Pumphrey, Scot H. Hulbert

**Affiliations:** Department of Plant Pathology, Washington State University, Pullman, WA 99164-6430 USA; Department of Crop and Soil Sciences, Washington State University, Pullman, WA 99164-6430 USA; Department of Biology, Carleton College, One North College St., Northfield, MN 55057 USA; US Department of Agriculture, Agricultural Research Service, Wheat Genetics, Quality, Physiology and Disease Research Unit, Pullman, WA 99164-6430 USA; US Department of Agriculture, Agricultural Research Service, Cereal Disease Laboratory, St. Paul, MN 55108 USA

**Keywords:** *Puccinia*, Haustorium, Host-induced gene silencing, Barley stripe mosaic virus, Virus-induced gene silencing

## Abstract

**Background:**

The cereal rust fungi are destructive pathogens that affect grain production worldwide. Although the genomic and transcript sequences for three *Puccinia* species that attack wheat have been released, the functions of large repertories of genes from *Puccinia* still need to be addressed to understand the infection process of these obligate parasites. Host-induced gene silencing (HIGS) has emerged a useful tool to examine the importance of rust fungus genes while growing within host plants. In this study, HIGS was used to test genes from *Puccinia* with transcripts enriched in haustoria for their ability to interfere with full development of the rust fungi.

**Results:**

Approximately 1200 haustoria enriched genes from *Puccinia graminis* f. sp. *tritici* (*Pgt*) were identified by comparative RNA sequencing. Virus-induced gene silencing (VIGS) constructs with fragments of 86 *Puccinia* genes, were tested for their ability to interfere with full development of these rust fungi. Most of the genes tested had no noticeable effects, but 10 reduced *Pgt* development after co-inoculation with the gene VIGS constructs and *Pgt*. These included a predicted glycolytic enzyme, two other proteins that are probably secreted and involved in carbohydrate or sugar metabolism, a protein involved in thiazol biosynthesis, a protein involved in auxin biosynthesis, an amino acid permease, two hypothetical proteins with no conserved domains, a predicted small secreted protein and another protein predicted to be secreted with similarity to bacterial proteins involved in membrane transport. Transient silencing of four of these genes reduced development of *P. striiformis* (*Pst*)*,* and three of also caused reduction of *P. triticina* (*Pt*) development.

**Conclusions:**

Partial suppression of transcripts involved in a large variety of biological processes in haustoria cells of *Puccinia* rusts can disrupt their development. Silencing of three genes resulted in suppression of all three rust diseases indicating that it may be possible to engineer durable resistance to multiple rust pathogens with a single gene in transgenic wheat plants for sustainable control of cereal rusts.

**Electronic supplementary material:**

The online version of this article (doi:10.1186/s12864-015-1791-y) contains supplementary material, which is available to authorized users.

## Background

The cereal rust fungi are destructive pathogens that affect global food security in spite of extensive and continual efforts on breeding, epidemiology and chemical control. Along with other biotrophic fungal and oomycete pathogens, like the powdery and downy mildews, rust fungi share a common infection process that involves the formation of haustoria within living plant cells. Haustoria are thought to play an essential role in nutrient uptake, redirection of host metabolism, and the suppression of host defenses [1]. Effector proteins of pathogenic microbes manipulate host metabolism and immunity [2, 3] and thus have received considerable attention. These effectors are usually secreted from pathogens to act at the host cell surface or inside host cells [4, 5]. Studies of biotrophic fungi have indicated that numerous effector proteins are expressed in haustoria and secreted into host cells and potentially recognized by resistance R proteins inside the host cells [3, 6–8]. Most of the effectors identified from biotrophic fungi have little or no homology to characterized proteins, so there are few clues to their functions. Furthermore, functional analysis is challenging due to the inherent difficulties with culturing and genetic transformation for these biotrophic pathogens.

Recent advances in the structural genomics of *Puccinia* species have shed light on various aspects of their biotrophic interactions with their cereal hosts. Draft genome sequences [9–11], along with transcript sequencing or transcript level analysis of genes expressed in colonized wheat plants are available for the three *Puccinia* spp. that attack wheat [10, 12–16]. The loss of pathways for nitrate and sulfate assimilation and expanded repertoire of amino acid and peptide transporters highlight the dependency of these fungi on host metabolism. Genes found to be up-regulated during plant colonization or, more specifically, in haustoria include those predicted to code for hydrolytic enzymes, energy production, and transport as well and many effector-like secreted proteins and other proteins of unknown function. Comparative analyses of rust fungi have indicated many of the latter proteins are specific to different rust fungus species [10, 17]. Associations between polymorphisms in genes encoding secreted proteins and virulence patterns in different isolates are being used to find candidates for effectors that interact with specific resistance genes in different wheat cultivars [12, 13].

Recently, host-induced gene silencing (HIGS) has emerged as an effective tool to characterize gene functions from biotrophic fungi [18, 19]. Panwar et al. [20] demonstrated that silencing three predicted pathogenicity genes (a MAPK, a cyclophilin and a calcineurin regulatory subunit) in *Puccinia triticina* (*Pt*) by HIGS could suppress leaf rust and also two other cereal rusts. In the barley-*Blumeria graminis* f. sp. *tritici* interaction, a class of ribonuclease-like effectors in the fungus was determined to contribute to infection by HIGS [3]. HIGS was also used to identify a tryptophan 2-monooxygenase gene involved in auxin biosynthesis from *Pgt* and demonstrate that it was required for full pathogenicity [21]. Additional technological advances include development of bacterial type III secretion systems to deliver fungal effector proteins into cereals [22, 23] which have been used to identify an effector from *Pgt* that induced a host-specific hypersensitive response (HR) in wheat [24]. While the field of biotrophic pathogen effector biology is moving rapidly, the functions of large repertories of candidate effectors and conserved proteins from biotrophic fungi still need to be addressed.

In this study, *Pgt* genes whose transcripts were enriched in haustorial cells and conserved among the three wheat-infecting *Puccinia* species were identified by comparative RNA sequencing and comparative genomics. A set of 76 *Pgt* genes and 10 *Pst* homologs were assayed for their importance in full development of these rust fungi. Ten of these were demonstrated to result in significant suppression of pathogen development after being silenced.

## Results

### Selection of candidate genes

Transcriptomes of *Pgt* infected wheat leaves and isolated haustoria were sequenced on an Illumina Genome Analyser GX II platform producing 28,558,894 and 16,000,237 raw reads from infected leaves and haustoria, respectively. After removing low quality and contaminating wheat sequences from the sequence data, this resulted in 13,073,158 and 10,663,092 reads for infected leaves and haustoria, respectively [Additional file 1]. Relative levels of transcripts of specific genes in haustorial cells versus all cell types in infected leaves were compared using the ratio of normalized sequence reads from a haustorial library compared to a library from infected leaves. A ratio of at least 2.0 was used to consider genes as likely haustoria-specific or haustoria-enriched and a total of 1182 such genes were identified. Several criteria were used to choose genes for silencing from the 1182 putative haustoria enriched genes while sampling a broad spectrum of types of genes. Preference was given to genes that had homologs in all three wheat-infecting *Puccinia* species with long stretches of DNA sequence identity, although four (PGTG_03590, PGTG_17724, PGTG_08762 and PGTG_09204) were specific to *Pgt.* Genes that were highly conserved in plants or mammals throughout their coding regions were avoided to minimize problems with silencing of non-target genes. Families of closely related genes were also avoided to limit confusion concerning which members were silenced in VIGS assays. A sample of genes were selected that were predicted to code for signal peptides and transmembrane domains using SignalP V4.0 to represent proteins that may directly interact with cellular components of host cells. Genes predicted to code for metabolic enzymes potentially involved in the synthesis of important cellular components (e.g. amino acids, lipids, etc.) and transporters involved in the uptake of essential compounds were also sampled. Based on these criteria, 86 of the 1182 genes were selected [see Additional file 2]. Thirty of the 86 genes were predicted to code for proteins with signal peptides and 12 for proteins with transmembrane domains. Thirty-seven of the genes were predicted to code for proteins of known function and were associated with eight general biological processes by using a BLAST2GO platform and manual annotation. Of these, 11 genes were predicted to be involved in transport processes, 11 genes were associated with carbohydrate metabolic processes, four were predicted to be involved in biosynthetic processes, three related to cellular lipid metabolism, three involved in mitochondrion organization, two associated with generation of precursor metabolites and energy, two related to gene expression, and one involved in cofactor metabolic processes. Most of the selected genes (49 genes) encoded predicted proteins of unknown function [Additional file 3].

### Host-induced gene silencing

To investigate whether the above selected fungal genes are important for pathogenicity or fungal development, silencing assays were conducted using BSMV-mediated HIGS. Our previous work focused mainly on silencing *P. striiformis,* where the transcript levels of seven genes with haustoria-enriched RNA levels were reduced but no obvious or repeatable effects on *Pst* development were observed [19]. From the 86 selected *Pgt* genes, *Pst* gene homologs (Additional file 2) were used for ten of them to assay their ability to suppress *Pst* development. The VIGS constructs were made as described previously [21] with cDNA fragments sizes of 252 bp to 476 bp inserted into the VIGS vector. For these *Pst* gene VIGS constructs, first and second leaves of 12-day-old wheat cultivar Zak were inoculated with the VIGS transcripts and infected plants then were challenged with *Pst* race PST-78 at 10 dpi. Although small reductions in *Pst* development were observed on some seedlings in some of the assays, none of the constructs had repeatable effects. In the hope that assays for silencing effects on development may be more repeatable or able to detect small effects with *Pgt* compared to *Pst*, the remainder of the genes (76 *Pgt* genes, Additional file 2) tested were *Pgt* genes and were first tested with a *Pgt* inoculations. The sizes of the *Pgt* cDNA fragments used for VIGS constructs ranged from 98 bp-500 bp.

**Fig. 1:**
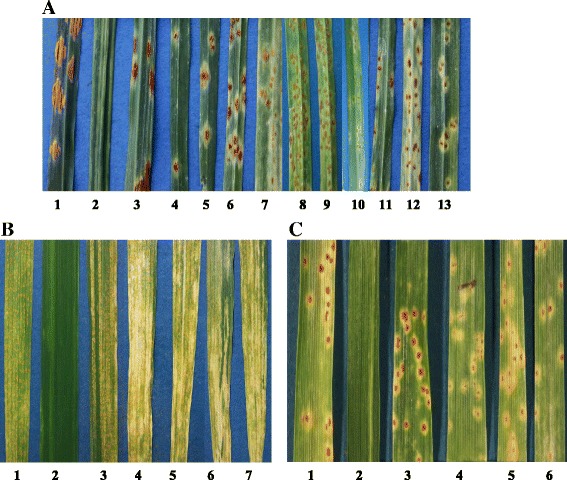
Reduced *Puccinia* spp. development on susceptible wheat after silencing. **a** Reduced *Pgt* (Pgt7A) development on wheat cultivar McNair 701. 1, Pgt7A without viral infection; 2, BSMV:MCS control virus inoculation without rust infection; 3, Pgt7A and BSMV:MCS infection; 4, Pgt7A and BSMV: *Pgt-IaaM*; 5, Pgt7A and BSMV:PGTG_01136; 6, Pgt7A and BSMV:PGTG_03590; 7, Pgt7A and BSMV:PGTG_01304; 8, Pgt7A and BSMV:PGTG_01215; 9, Pgt7A and BSMV:PGTG_03478; 10, Pgt7A and BSMV:PGTG_10731; 11, Pgt7A and BSMV:PGTG_12890; 12, Pgt7A and BSMV:PGTG_14350; 13, ‘Sr31/6*LMPG’ wheat carrying resistance gene *Sr31* challenged with Pgt7A. Pictures were taken at 12 dpi. **b** Reduced *Pst* (PST-78) development on wheat cultivar Zak after silencing. 1, PST-78 without viral infection; 2, BSMV:MCS inoculation without rust infection; 3, PST-78 and BSMV:MCS infection; 4, PST-78 and BSMV: PSTG_04507; 5, PST-78 and BSMV: PSTG_03360; 6, PST-78 and BSMV: PSTG_04871; 7, PST-78 and BSMV: PSTG_11830. Pictures were taken at 18 dpi. **c** Reduced *Pt* development on wheat cultivar McNair 701 after silencing. 1, *Pt* without viral infection; 2, BSMV: MCS inoculation without rust infection; 3, *Pt* and BSMV: MCS infection; 4, *Pt* and BSMV: PSTG_04507; 5, *Pt* and BSMV: PSTG_03360; 6, *Pt* and BSMV: PSTG_04871. Pictures were taken at 12 dpi

To test the *Pgt* gene VIGS constructs, first and second leaves of 12-day-old seedlings of *Pgt*-susceptible wheat cultivar McNair 701 were rub inoculated with the transcripts from either the VIGS construct that carried a candidate gene target region or the control virus consisting of the same vector without the fungal DNA fragment incorporated into the multiple cloning site of the γ genome. After 10 days, infected plants displaying mild systemic mosaic viral symptoms were challenged with *Pgt* isolate CDL 75-36-700 (Pgt7A). The infected plants were kept in a growth chamber and the infection types (IT) were scored at 12 days post inoculation (dpi) using the 0-to-4 scale similar to that described by Stakman et al. [25] to reflect uredinia size and abundance. Briefly, 0 and 1 ITs have no uredinia or just a few, respectively, and were generally not observed in our VIGs assays. Infection types of 2 clearly have much smaller uredinia than fully susceptible ITs, while ITs of 3 have smaller uredinia than fully susceptible plants but are intermediate between 2 and 4 ITs. Twelve seedlings were used for each VIGS construct in each assay. Assays of VIGS constructs that showed seedlings with reduced rust development were repeated at least four times. VIGS assays with ten genes consistently showed seedlings with a reduction of *Pgt* development (Table 1). In each assay with these 10 VIGS constructs, seedlings with reduced IT values of 2 and 3 were apparent (Fig. 1a) as well as seedlings with ITs of 4. The control plants inoculated with BSMV: MCS (or controls with no virus infection) consistently displayed susceptible ITs of 4. Thus even though the VIGS assays consistently showed many fully susceptible seedlings, those with lower infection types were consistently observed in assays with the ten *Pgt* genes and never observed with the control VIGS constructs without the gene fragments. The frequency that lower infection types were observed ranged from 6 % for PGTG_03478 to 20 % for PGTG_12890 (Table 1).

**Table 1:** Summary of ten *Puccinia graminis* f. sp. *tritici* genes contributing to pathogenicity or development

Gene ID	Putative gene functions	Transcript levels^a^	SP^b^	Homolog similarity (nt identities %)		Homolog similarity (aa identities %)		Frequency of low ITs^c^
		Haust/Infw		*Pt*	*Pst*	*Pt*	*Pst*	
PGTG_01136	Fructose-bisphosphate aldose	5.80	No	89	86	91	81	14/164
PGTG_01215	Glycoside hydrolase family 26	5.8	Yes	83	83	80	80	22/131
PGTG_03478	Glycoside hydrolase family 76	7.3	Yes	N/A	N/A	52	53	9/143
PGTG_14350	Transporter component-like	2.4	Yes	86	82	89	88	25/154
PGTG_10731	Predicted protein	6.6	No	85	81	91	85	22/132
PGTG_12890	Predicted protein	7.1	No	78	81	82	80	32/160
PGTG_01304	Thiazole biosynthetic enzyme	2.1	No	91	88	95	96	23/127
PGTG_16914	Amino acid transporter	15.4	No	86	85	83	83	7/69
PGTG_03590	Small secreted protein	81.8	Yes	N/A	N/A	N/A	N/A	15/159
*Pgt-IaaM*	Tryptophan 2-monooxygenase	11.5	No	80	83	82	79	13/141

^a^Frequency of fungal RNA sequence reads for the transcript in isolated haustoria of infected plants over the frequency from RNA from infected leaves

^b^ *SP* predicted signal peptide

^c^Number of seedlings showing reduced infection types/number of seedlings used in VIGS assays. Data are pooled from four or more experiments

Genomic DNA was extracted from seedling leaves used in one of the VIGS assay experiments to quantify the amount of fungus present in the wheat leaves and determine if the seedlings with lower ITs also had less fungal biomass in the leaves at 12 dpi. Wheat seedlings were inoculated either with the VIGS vector carrying no fungal sequences as a control or one of the VIGS constructs from each of the ten *Pgt* genes. Quantitative PCR was used to estimate the ratio of the *Pgt* DNA to wheat DNA of the infected seedling leaves. As with the other VIGS assays with these genes, seedlings inoculated with each of the VIGS constructs showed both fully susceptible seedlings (IT = 4) and seedlings with lower ITs (IT = 2 to 3) while seedlings infected with the control vector all showed ITs of 4. Although the ratio of fungal to plant DNA was somewhat variable due to variation in inoculation coverage and infection of the individual leaves, those with lower ITs (infected with *Pgt* gene VIGS constructs) had significantly (P < .0001) lower ratios of fungal DNA to plant DNA than those with higher ITs (infected with control or Pgt gene VIGS constructs). Leaves with low infection types (pooled from each of the 10 VIGS constructs) had an average ratio of fungal to plant DNA of 3.81 ± 2.92 (n = 34) while those infected with the same VIGS constructs but infection types of 4 had an average of 9.80 ± 6.29 (n = 28) and seedlings infected with the control virus had a ratio of 11.96 ± 5.15 (n = 36). As expected, the poorer uredinia development in seedling leaves with the lower ITs also generally had lower levels of fungal biomass at the onset of sporulation.

The ten *Pgt* genes that suppressed disease development included: a predicted glycolytic enzyme (PGTG_01136); two proteins (encoded by PGTG_01215 and PGTG_03478) probably involved in cellular carbohydrate or sugar metabolism; a hypothetical secreted protein with homology to periplasmic components of prokaryotic transport systems (PGTG_14350); two hypothetical proteins with no conserved domains and homology to genes in a variety of basidiomycetes (PGTG_10731 and PGTG_12890); a protein involved in thiazol biosynthesis (PGTG_01304); an amino acid permease (PGTG_16914); a small secreted protein (PGTG_03590); and a tryptophan 2-monooxygenase enzyme (PGTG_11658, designated *Pgt-IaaM*) that several plant pathogenic bacteria and a few fungi use to make the IAA precursor indole-3-acetamide [21] (Table 1).

### Host induced silencing effects across *Puccinia* species

Sequence comparisons using BLAST revealed that eight of these ten *Pgt* genes (except for PGTG_03478 and PGTG_03590) were highly conserved in their nucleotide sequences in the three wheat rust fungi, and also had clear homology to genes in *Melampsora larici-populina* (Table 1). The PGTG_03590 gene had no homologous sequences in the *Pst* or *Pt* databases and PGTG_03478 showed much less homology to the others with only approximately 50 % predicted amino acid identity to the closest *Pt* (PTTG_03623) and *Pst* (PSTG_15691) sequences. The gene PGTG_03478 belongs to a gene family in *Pgt* with at least seven homologs, which ranged from 40 % to 68 % amino acid identity to PGTG_03478. The *Pst* and *Pt* genomes carry four and nine homologs, respectively. The *Pst* (PSTG_15691) and *Pt* (PTTG_03623) genes show higher homology (80 % and 75 % amino acid identity) to one of the other *Pgt* family members (PGTG_03483) indicating that the genes orthologous to PGTG_03478 may have been lost in their genomes. Alternatively the apparent differences between the gene families in the three *Puccinia* species could be due to incomplete genome assemblies or due to gene expansions that occurred after speciation. Low levels of DNA sequence homology and lack of clear orthologs precluded the use of the PGTG_03478 and PGTG_03590 gene sequences for silencing experiments in *Pst* and *Pt*.

To test whether the eight highly conserved genes have similar roles in fungal development in the different *Puccinia* species, silencing experiments were conducted with *Pst* and *Pt*. The first and second leaves of 12-day-old wheat cultivar Zak were inoculated with the transcripts produced from the eight *Pgt* gene VIGS constructs, and infected plants then were challenged with PST-78 at 10 days after virus inoculation. After 18 days, lower IT values of 4 to 6 (0 to 9 scale) were observed in many of the wheat leaves infiltrated with four of the target VIGS constructs (PGTG_01136, *Pgt-IaaM*, PGTG_01215 and PGTG_12890), compared with control plants inoculated with BSMV:MCS or no BSMV which consistently displayed susceptible ITs of 7 to 9. The remaining four genes did not display noticeable suppression of *Pst* development, and these were not examined further. To confirm these results, VIGS constructs of the four genes were made using the homologous *Pst* sequences of these four genes (PSTG_04507, PSTG_03360, PSTG_04871 and PSTG_11830). Larger gene fragments were used when making the constructs from *Pst* sequences [Additional file 4] with the hope that they would more efficiently silence homologous sequences in related rust species. Similar phenotypes, lower IT values of 4 to 6, were observed in wheat leaves infiltrated with four target constructs (Fig. 1b). Seedlings with lower infection types were observed in 22 of 83 seedlings tested for PSTG_04507, 20 of 80 for PSTG_03360, 19 of 66 for PSTG_04871 and 24 of 96 for PSTG_11830. To determine if these genes were also important for *Pt* fungal development, wheat leaves infected with the above *Pst* VIGS constructs were challenged with *Pt* urediniospores known to infect wheat cultivar McNair701. After 12 days, the leaf rust IT values of 2 to 2+ (0 to 4 scale) were observed in wheat leaves infiltrated with three target VIGS constructs, compared with consistently susceptible ITs in the control plants (Fig. 1c). The PSTG_04507, PSTG_03360 and PSTG_04871 constructs provided fungal development suppression but the phenotype of the construct for PSTG_11830 was similar to the control plants. Lower infection types were observed in 10 of 58 seedlings for PSTG_04507, 14 of 64 for PSTG_03360 and 14 of 53 for PSTG_04871. Overall, the results indicated that these three genes (PGTG_01136, *Pgt-IaaM* and PGTG_01215) contributed to full colony development of *Pgt, Pst* and *Pt* and are important for pathogenicity for all three wheat rust fungi.

The effect of the infection with the VIGS constructs on the transcript levels of the ten genes affecting *Pgt* colony development was examined by RT-qPCR assays (Table 2). The relative expression of the *Pgt*, *Pst* or *Pt* genes in individual seedlings infected with the various VIGS constructs were compared to the expression of the same genes in six different control seedlings infected with the VIGS vector without fungal sequences inserted. As was observed in a previous analysis with *Pst* gene silencing experiments (Yin et al. 2011), reductions in transcript levels were sometimes small or not reduced and occasional seedlings showed increased levels compared to the controls. However, assays with all of the genes identified seedlings with reduced transcript levels but the level of reduction was moderate (<50 %) for some of the genes. The variability in silencing of fungal genes in the VIGS assays is probably partly responsible for the low frequencies of seedlings with low IT scores (Table 1).

**Table 2:** Relative transcript levels of *Puccinia* genes in wheat seedlings infected with VIGS constructs compared to seedlings infected with the control BSMV virus

Gene ID	Relative transcript levels of target gene in HIGS assays^a^							*Puccinia* spp. Tested^b^
	Leaf 1	Leaf 2	Leaf 3	Leaf 4	Leaf 5	Leaf 6	Average^c^	
PGTG_03590	0.55 ± 0.10	0.46 ± 0.09	0.32 ± 0.06	0.36 ± 0.07	0.49 ± 0.10	0.37 ± 0.08	0.425 ± 0.09	*Pgt*
PGTG_01136	0.79 ± 0.34	0.65 ± 0.27	0.25 ± 0.11	0.40 ± 0.17	0.67 ± 0.28	0.76 ± 0.32	0.59 ± 0.21	*Pgt*
PGTG_01215	0.47 ± 0.18	0.49 ± 0.19	0.36 ± 0.14	0.82 ± 0.32	0.58 ± 0.22	3.01 ± 1.16	0.96 ± 1.02	*Pgt*
PGTG_12890	0.21 ± 0.04	0.72 ± 0.14	0.77 ± 0.14	0.45 ± 0.09	0.54 ± 0.11	0.52 ± 0.10	0.54 ± 0.20	*Pgt*
PGTG_03478	0.59 ± 0.26	0.14 ± 0.06	0.72 ± 0.31	0.65 ± 0.28	0.81 ± 0.35		0.58 ± 0.26	*Pgt*
PGTG_10731	0.68 ± 0.14	1.44 ± 0.29	0.57 ± 0.12	0.68 ± 0.14	1.33 ± 0.27	2.45 ± 0.50	1.19 ± 0.72	*Pgt*
PGTG_14350	0.25 ± 0.08	0.62 ± 0.21	0.13 ± 0.04	0.25 ± 0.09	0.74 ± 0.25	1.96 ± 0.67	0.66 ± 0.68	*Pgt*
PGTG_01304	0.43 ± 0.09	0.76 ± 0.16	0.73 ± 0.15	0.37 ± 0.08	0.84 ± 0.18	0.64 ± 0.13	0.63 ± 0.19	*Pgt*
PSTG_04507	0.33 ± 0.18	0.23 ± 0.13	0.01 ± 0.01	0.40 ± 0.22	0.72 ± 0.40	3.54 ± 1.96	0.87 ± 1.33	*Pst*
PSTG_11830	0.58 ± 0.08	0.63 ± 0.09	0.38 ± 0.05	1.39 ± 0.19	0.95 ± 0.13	0.92 ± 0.13	0.81 ± 0.36	*Pst*
PSTG_04871	0.68 ± 0.10	0.87 ± 0.13	0.41 ± 0.06	0.69 ± 0.11	0.40 ± 0.06	1.64 ± 0.25	0.78 ± 0.46	*Pst*
PSTG_03360	0.76 ± 0.16	0.68 ± 0.14	0.67 ± 0.14	0.71 ± 0.15	0.57 ± 0.12	1.02 ± 0.22	0.74 ± 0.15	*Pst*
PSTG_04507	0.28 ± 0.26	0.46 ± 0.41	0.05 ± 0.04	0.58 ± 0.52	0.19 ± 0.18	0.26 ± 0.24	0.30 ± 0.19	*Pt*
PSTG_04871	0.57 ± 0.09	0.66 ± 0.11	0.71 ± 0.12	0.74 ± 0.12	0.71 ± 0.11	0.61 ± 0.10	0.67 ± 0.07	*Pt*
PSTG_03360	0.67 ± 0.35	0.65 ± 0.34	0.41 ± 0.21	0.10 ± 0.05	0.62 ± 0.32		0.49 ± 0.24	*Pt*

^a^Values represent the average ratio of transcript levels from the VIGS challenged seedling leaf to transcript levels in six individual seedlings infected with the control virus, and the standard deviation of these six ratios

^b^PGTG genes were infected with *Pgt* and *Pgt* transcript levels were measured. PSTG genes were infected with either *Pst* or *Pt*

^c^Average and standard deviation of the estimates of relative transcript levels of each of the individual plants in one HIGS assay

The effect of the PGTG-Iaam construct on gene expression was examined previously [21]

### Transcription patterns of promising HIGS target genes

**Fig. 2:**
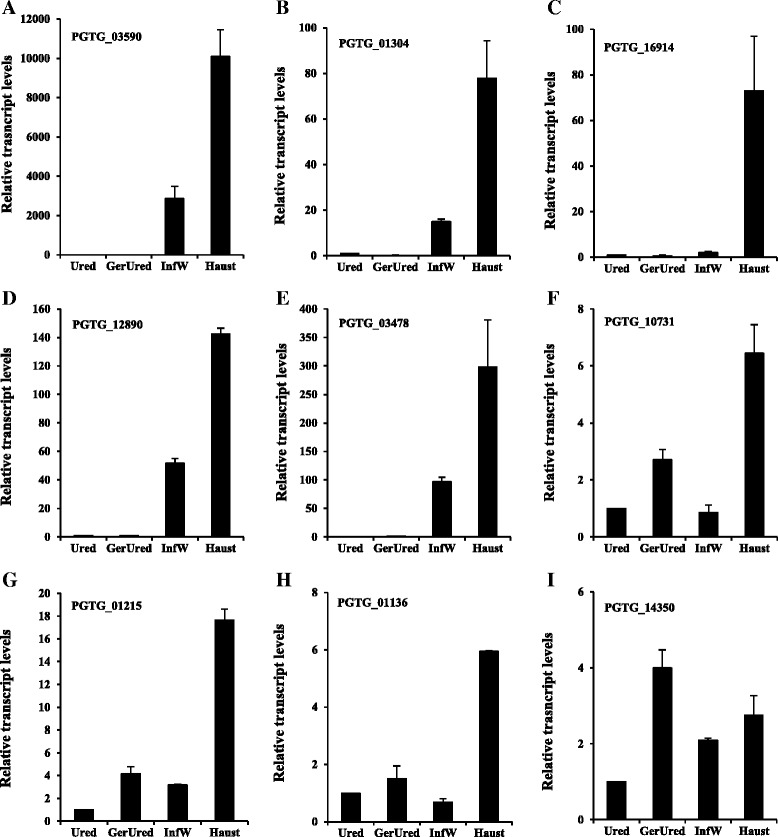
Gene transcript levels in different developmental stages in *Puccinia graminis* f. sp. *tritici,* relative to transcript levels in urediniospores. Panels **a** to **i** correspond to transcript levels of nine different P g t genes as indicated in the panels. Ured: urediniospores; GerUred: germinated urediniospores; InfW: infected wheat leaves; and Haust: isolated haustoria. Standard deviations were calculated from values obtained from three technical replicates. Similar results were obtained for each of two biological replicates

The RNA sequencing transcript analysis used to select *Pgt* genes was performed on only a single replication of infected leaves and isolated haustoria and therefore a potentially inaccurate measure of transcript levels. To reexamine the transcript patterns of the ten genes which contribute to *Pgt* development, RT-qPCR analysis was conducted. Transcript levels were monitored in total RNA extracted from *Pgt* urediniospores, urediniospores germinated *in vitro*, infected wheat leaves at 5 dpi (before sporulation was observed) and isolated haustorial cells. The transcript levels in each tissue were normalized relative to the transcript levels of the *Pgt* Actin gene [26]. The specific RNA content was then calculated relative to the abundance of the RNA of the candidate gene in urediniospores. The degree to which the transcript levels were specific to infected plants or haustoria varied greatly. The transcript levels of PGTG_03590, PGTG_01304, PGTG_16914, PGTG_12890 and PGTG_03478 all showed an increase in infected leaves compared with urediniospores and even higher levels in isolated haustoria (Fig. 2a-e). *Pgt-IaaM* transcript profiles by RT-qPCR have been described previously and appeared similar; transcript levels in haustoria were approximately seven times higher than in infected plants and several hundred times higher than urediniospores [21]. The accumulation of PGTG_10731, PGTG_01215 and PGTG_01136 transcripts increased in germinated urediniospores compared to urediniospores, but also showed higher levels in isolated haustorial than in infected leaves (Fig. 2f-h). PGTG_14350 transcript levels showed the least tissue specificity of the ten genes. They showed an increase of four-fold in germinated urediniospores compared to non-germinated urediniospores, and only a slight increase (1.3-fold) in haustoria compared to infected plants. Transcript levels for PGTG_14350 in isolated haustoria were also predicted to be only 2.4-fold higher than whole infected leaves by the transcript sequencing analysis (Fig. 2i). The decreased *Pgt* development in assays with PGTG_14350 implies that a high level of haustoria specificity is not necessarily required to interfere with *Puccinia* development. It is also apparently not necessary for the genes to be transcribed at high levels in haustoria since the 10 genes that interfered with *Pgt* development showed a large range of transcript levels. The PGTG_01304 gene transcript levels were very high in haustorial cells and were identified in the RNA sequencing experiment in 5354 reads per kb per million total sequence reads (RPKM). In contrast, the PGTG_03478 and PGTG_12890 transcripts levels were 10 and 19 RPKM. The remaining genes showed more intermediate transcript levels: PGTG_IaaM = 45; PGTG_01215 = 311; PGTG_10731 = 329; PGTG_14350 = 335; PGTG_03590 = 436; PGTG_01136 = 1131, and PGTG_16914 = 1173 RPKM.

The observation that the gene coding for the glycolytic enzyme fructose bisphosphate aldolase (PGTG_01136) showed a 6-fold increase in transcript levels in haustoria raises the question of whether the whole glycolytic pathway is up-regulated in haustoria. Interestingly, the preliminary RNA sequence data showed an increase transcript levels in isolated haustoria compared to infected leaves for the 10 genes encoding all of the glycolytic enzymes (Table 3). Similar observations were made for *Pgt,* comparing haustoria expression to germinated spores [27], and also in *Pst* [14] and the other haustoria-forming obligate pathogens *U. fabae* [28] and *Blumeria graminis* [29]. This indicates that this process is generally very active in haustoria during infection. To determine if silencing the other genes coding for glycolytic enzymes in haustoria was detrimental to fungal development, VIGS constructs were made from sequences of *Pgt* genes coding for the other nine enzymes (Table 3). When seedlings of the wheat cultivar McNair 701 were challenged with these nine VIGS constructs and *Pgt* isolate Pgt7A, no significant reduction in fungal development was observed compared with the controls.

**Table 3:** Relative transcript levels of genes encoding glycolytic enzymes in *Puccinia striiformis* f. sp. *tritici*

Gene ID	Putative gene functions	Transcript levels
		Haust/Infw^a^
PGTG_18333	Hexokinase	2.9
PGTG_07534	Glucose-6-phosphate isomerase	2.1
PGTG_05736	6-phosphofructokinase	3.7
PGTG_01136	Fructose-bisphosphate aldolase, class ii	5.8
PGTG_00038	Triosephosphate isomerase	4.2
PGTG_04956	Glyceraldehyde-3-phosphate dehydrogenase	4.3
PGTG_17029	Phosphoglycerate kinase	2.9
PGTG_04139	phosphoglycerate mutase	122.5
PGTG_14903	Enolase	3.3
PGTG_16473	Pyruvate kinase	2.6

^a^Frequency of fungal RNA sequence reads for the transcript in isolated haustoria of infected plants over the frequency from RNA from infected leaves

## Discussion

The currently available genomic and transcript sequences for three *Puccinia* species present opportunities to examine various biological processes. Partial inactivation of genes by HIGS has potential for examining the importance of rust fungus genes with unknown functions while growing within host plants [18, 19]. Previous work indicated transcript levels were reduced most efficiently in haustorial cells of *Pst* by HIGS [19]. So genes whose transcripts in infected plant tissue were abundant in haustoria cells were the focus of this study. Eighty-six *Puccinia* haustoria enriched genes, representing a variety of biological processes, were analyzed for their ability to interfere with fungal development using BSMV-based HIGS. Silencing 10 *Pgt* genes suppressed full fungal development in highly susceptible wheat cultivars indicating pathogenicity or other aspects of development were compromised. The genes were predicted to be involved in a variety of known processes, including carbohydrate metabolism, nutrient transport, and hormone and vitamin biosynthesis, as well as unknown processes. The majority of the genes silenced did not noticeably interfere with fungal development. This should not be taken as evidence that their transcription in haustorial cells is not important but simply that no evidence was found that it is required. It is possible that silencing was not sufficiently complete to knock down levels of encoded proteins to levels that would interfere with fungal development. It is also possible that the genes are sufficiently expressed in adjacent un-silenced cells and their expression is sufficient to supply the haustoria with the essential function. Genetic or functional redundancy is another possibility for not observing a phenotype after silencing. Several of the genes targeted were members of gene families with two or more members, including three genes predicted to code for transporters and 15 secreted proteins. Interestingly, most of the haustorium-specific *Pgt* genes encoding secreted proteins that were conserved among the three *Puccinia* species were also found to be members of gene families with two to approximately 40 members.

Haustorial cells are thought to play the primary role in nutrient acquisition from the host for biotrophic fungi growth. Genes selected for silencing included three predicted amino acid transporters (PGTG_16914, PGTG_07026 and PGTG_00315) and two sugar transporters (PGTG_18584 and PGTG_21065) and seven other genes predicted to encode transporter-like proteins. Of these, only two genes appeared important to fungal development in our silencing assays, one of which was the predicted amino acid transporter (PGTG_16914). This gene was homologous (82 % amino acid identity) to an *Uromyces fabae* amino acid transporter (PIG2, also designated AAT2) specific to haustorial membranes [30, 31]. As in the bean rust fungus, the transcript was abundant in *Pgt* accounting for approximately 0.25 % of the RNA sequence reads in isolated haustorial RNA. Garnica et al. [14] also found an AAT2 homolog in *Pst* was very highly transcribed in haustoria compared to germinated spores. The other two amino acid transporters, PGTG_00315 and PGTG_07026, were expressed at lower levels, with approximately 0.04 % and 0.01 % of the sequence reads, respectively. The predicted protein from the PGTG_00315 gene was 80 % identical to that of the *U. fabae UfAAT3* gene whose transport function was demonstrated with a *Xenopus* oocyte system [32]. The PGTG_14350 gene also appeared important in silencing assays and may be involved in transport. It is predicted to code for a secreted protein with homology to uncharacterized proteins in a large variety of fungi and weaker homology to periplasmic components of bacterial transport systems.

Glycoside hydrolases (GHs) are enzymes that cleave the glycosidic bond between carbohydrates or between carbohydrate and noncarbohydrate moieties. GHs are classified into more than 100 families in the Carbohydrate Active enZYmes (CAZy) database [33, 34]. In this study, four genes were tested that were predicted to encode for glycoside hydrolases that are potentially secreted from haustorial cells [see Additional file 2]. Two of these genes affected fungal development when silenced, PGTG_01215 and PGTG_03478, which encode glycoside hydrolase families 26 and 76, respectively. Enzymes in these families are primarily considered as mannanases [35]. Both PGTG_01215 and PGTG_03478 are members of small gene families whose members have diverged in sequence but were chosen for analysis because they were highly up-regulated in haustoria (Table 1 and Fig. 2g, 2e). These proteins may be secreted to make sugars available from storage mananns but other roles, such as hydrolyzing host cell wall components, are also possible. Silencing of PGTG_00004 and PGTG_15914, predicted to encode a family 3 and family 18 GHs, respectively, showed no suppression in fungal development in the VIGS assays. The enzymes avenacinase and tomatinase of phytopathogenic fungi, which belong to the GH3 class, deglycosylate and detoxify saponins of the host plants during infection [36, 37]. Other GHs implicated in pathogenicity include the GH10 and GH11 endoxylanases which play important roles in both vertical penetration and horizontal expansion of *M. oryzae* in infected leaves [38]. Disruption of multiple genes encoding GH6 and GH7 by RNAi gene silencing also resulted in reduced virulence of *M. oryzae* [39].

Genes encoding non-secreted enzymes involved in carbohydrate or sugar metabolism within fungal cells may appear important in silencing assays if these processes are required within the haustorial cells. One such gene is PGTG_01136 which encodes a fructose-1, 6-bisphosphate aldolase (FBA), a key enzyme of glycolytic pathway that performs the reversible cleavage of fructose 1, 6- bisphosphate (FBP) to glyceraldehyde 3-phosphate and dihydroxyacetone phosphate [40]. While the other glycolytic enzymes show homology between kingdoms, two distinct classes of FBAs occur. Class I is found in animals, plants and other higher organisms [41]. Class II is commonly present in lower organisms [42], like bacteria and fungi (including *Puccinia* species). The sequences of the two classes of FBAs are distinct and their catalytic mechanisms are also different [43]. For these reasons, it has been suggested that FBA could be a good drug or vaccine target against microbial pathogens [44–47]. Examination of the *Puccinia* sequences in databases indicates FBA is encoded by one gene in the *Pgt* and *Pst* genomes and two genes in the *Pt* genome but all shared high levels of sequence homology (89 % aa identity). Our transient silencing results showed significant disease suppression in all three *Puccinia* species tested, indicating that it is a good target to generate broad-spectrum genetic resistant against multiple cereal rust fungi for all three species. Genes for the other glycolytic enzymes were also up-regulated in haustorial cells compared to their transcript levels in most other fungal cells in infected wheat plants (Table 2). Similar up-regulation has been observed before in rust fungi [14, 27] and other haustoria forming fungi [28, 29] implying that glycolysis is an important process in haustorial cells. However, attempts at silencing the other nine genes encoding *Pgt* glycolytic enzymes showed no evidence of reductions in fungal development. It is possible that these other glycolytic genes are less sensitive to perturbations in transcript levels and therefore did not disrupt the glycolytic pathway. Alternatively the reaction catalyzed by FBA, transitioning between six and three carbon compounds may be particularly important in haustorial cells. The potentially greater effectiveness of FBA and its lack of homologs in plants and animals may make it a more suitable target for RNA interference approaches to engineer resistance to rust pathogens.

The PGTG_01304 gene appeared important for full colony development in our silencing assays and showed high transcript levels in haustorial cells, accounting for approximately 0.8 % of the RNA sequence reads from isolated haustoria. It is highly homologous (91 % aa identity) to the bean rust fungus *U. fabae* gene (*THI4 _UROFA*) [48, 49] and 89 % aa identical to *M. larici-populina* gene *Mlp*-53832 [10], both encoding a thiazole biosynthesis enzymes that are involved in thiamine synthesis and shown to be highly up-regulated during the interaction with their host plants. Garnica et al. [14] also observed relatively high levels of transcripts of a homologous *Pst* gene in haustoria, as well as transcripts of other genes involved thiamine biosynthesis. The results suggest that high levels of thiamine, or its derivatives, are required in haustorial cells. Thiamine pyrophosphate is a cofactor required for the activity of several enzymes in central carbon metabolism. It is interesting that a homologous rice gene is up-regulated after pathogen infection and is part of an important defense response [50]. It seems that both the pathogen and host need high levels of thiamine in cells involved in the interaction for pathogenicity and defense, respectively.

Four other genes appeared important in silencing assays, including a previously described *Pgt*-*IaaM* gene, a gene (PGTG_03590) encoding a *Pgt*-specific small secreted protein (162 aa) and two genes (PGTG_10731 and PGTG_12890) encoding proteins with predicted transmembrane domains but no other characterized domains. The two predicted transmembrane proteins had homologs in other rust fungi and a variety of other basidiomycetes, but none of these have characterized functions.

Understanding the importance of rust fungus genes in pathogenicity could assist development of resistant cultivars in multiple ways. As methods become developed to identify host R gene interactions with known effectors, information concerning the importance of the effectors, or the diversity of processes they are involved in, could assist selection of specific R genes to increase chances of developing durable resistance. More direct utilization of the gene sequences in cultivars by engineering HIGS may have great potential. While our transient assays generally provided modest levels of fungal development suppression, this should be assayed in transgenic lines that stably express the siRNAs. Silencing of three of the genes resulted in suppression of all three rust diseases: one encoding an enzyme involved in glycolysis (PGTG_01136), a secreted glycoside hydrolase and an enzyme involved in IAA biosynthesis. Suppression of multiple rusts by silencing a single target gene was also observed by Panwar et al. [20]. The potential for generating durable resistance to multiple rusts in a single transgene construct should warrant further research in HIGS approaches for sustainable control of cereal rusts.

## Conclusions

Ten of 86 genes with transcripts enriched in haustoria interfered with *Pgt* development when their transcripts were reduced in HIGS assays. The 10 genes were predicted to be involved in multiple biological processes in fungi. Engineering plants to silence multiple transcripts of essential genes involved in multiple processes will likely provide a higher level of disease suppression and should be more difficult for the pathogen to overcome. Three of the ten genes were sufficiently conserved in sequence and function to affect development in all three *Puccinia* rust fungi tested, but had no homologs in plants or animals. Therefore, these sequences may also be useful for engineering rust resistance in other grain and forage crops that are attacked by *Puccinia* rust fungi.

## Methods

### Plants materials, fungal races and growth conditions

The plants used in this study were wheat cultivars McNair 701, Zak and Sr31/6*LMPG. McNair 701 was used for *Pgt* and *Pt* gene-silencing assays, Sr31/6*LMPG with resistance *Sr31* was used as a stem rust resistant control, Zak was used for *Pst* gene-silencing assays. Wheat seedlings for gene-silencing assays were sown in pots containing potting mix and placed in growth chambers as previously described [19]. Urediniospores of *Pgt* isolate CDL 75-36-700 (Pgt7A) on McNair 701 and an uncharacterized isolate of *Pt* collected from Washington State in 2013 and *Pst* race PST-78 were increased on Zak.

To isolate fungal RNA from different infection stages for transcript analysis, urediniospores were harvested from infected McNair 701 leaves at 12-14 dpi with Pgt7A. Fresh urediniospores were either frozen, germinated by floating on sterile distilled water in glass petri dishes by incubation in the dark for 15 h at room temperature, or used to inoculate wheat seedlings. Infected wheat leaves were harvested at 5 dpi with Pgt7A (prior to sporulation). Haustoria were isolated from heavily infected wheat leaves at 5 dpi using ConA affinity chromatography as previously described [16]. Urediniospores, germinated urediniospores, infected wheat leaves and haustoria were stored at -80 °C for RNA extraction.

### Sequences from *Pgt*-infected wheat leaves and haustorial libraries and sequence analysis

Total RNA was extracted from *Pgt*-infected (five dpi) wheat leaves and isolated haustorial cells using the Qiagen Plant RNeasy kit (Qiagen, Chatsworth, GA) according to the manufacturer’s instruction. The quantity of isolated total RNA was checked for integrity by 2 % agarose gel electrophoresis as well as on an Agilent 2100 Bioanalyzer. RNA sequencing was performed by the National Center for Genome Resources using an Illumina Genome Analyzer GX II platform. Sequence lengths of 76 bases were generated for paired ends of transcripts. The wheat sequences were first filtered out by using the Wheat GI cDNA data set v 12.0. The remaining reads were assembled *de novo* and the resulting contigs were used in homology searches of the three *Puccinia* spp. genome database using the BLASTX and BLASTN algorithm. Haustorium specific or enriched genes were identified by comparing normalized numbers of sequence reads (reads per million) in isolated haustorial cells to transcript levels in all cell types in infected plant leaves. Genes with at least twice the frequency of sequence reads in transcripts from haustorial preparations as infected plant preparations were considered as potentially haustoria specific or haustoria enriched. The predicted proteins were then used in homology searches (BLASTP) with the NCBI and Broad Institute *Puccinia* databases to determine genomic copy number in *Pgt*, potential homologs in various taxa and insight into possible functions. Putative signal peptides and transmembrane domains in the deduced amino acid sequences of candidate genes were predicted using SignalP V4.0 (http://www.cbs.dtu.dk/services/SignalP-4.0/). The candidate genes were used to conduct GO functional analysis using BLAST2GO (B2G) [51, 52]. Additional annotation was conducted by manually examining data collected on homologous genes from other organisms.

### Construction of BSMV-derived vector and *in vitro* transcription of viral RNAs

Target gene nucleotide sequences were used to search the GrainGenes database http://wheat.pw.usda.gov/GG3/ to avoid homology with wheat or barley sequences. Most of the genes selected for silencing had no obvious homologs to plant or mammalian sequences although some coded for conserved enzymes and some had short stretches of perfect homology to cereal sequences by chance. Highly conserved regions of these genes and any sequences with stretches of more than 19 nucleotides of perfect homology to cereal genes were avoided. Primers were designed (listed in Additional file 4: Table S3) to amplify fragments of approximately 100-500 nt in the initial constructs made for silencing a single rust species. Larger fragments (400-600 nt) were used for some of the gene fragments tested on multiple *Puccinia* species. The amplicons were double digested with *Not*I and *Pac*I and directionally ligated into *Not*I*/Pac*I sites of the BSMV γ vector so that the viral genome carried the antisense strand of the target gene. The derived pγ construct, pα, and pβ Δβa were linearized by *Bss*HII, *Mlu*I or *Spe*I digestion, respectively. *In vitro* transcripts were prepared from the three linearized plasmids using the mMessage mMachine T7 *in vitro* transcription kit (Ambion, Austin, TX, U.S.A.) following the manufacturer’s instructions. The negative control (BSMV: MCS) carried only a 121-bp fragment of the multiple cloning sites (MCS) from pBluescript K/S and carried no *Puccinia* spp. sequences [19].

### Virus and *Puccinia* inoculations and rust assays

The first and second fully expanded leaves of 12-day-old wheat McNair 701 or Zak were inoculated with the transcripts produced from the BSMV construct carrying the target gene fragment. By 10 dpi, when virus symptoms became apparent on newer non-inoculated leaves, only those leaves displaying mild virus symptoms were inoculated with urediniospores of Pgt7A, PST-78 or *Pt*. Samples of the infected leaves were harvested for RNA extraction at 5 dpi for *Pgt* and *Pt*, and 8 dpi for *Pst*. The remaining infected plants were kept in the growth chamber until 12 dpi for *Pgt* and *Pt*, 18-20 dpi for *Pst*. Infection types (ITs) were assessed based on the 0-to-4 rating scale [25] for *Pgt* and *Pt*, and on the 0-to-9 scale [53] for *Pst*. The BSMV: MCS vector construct, with no inserted sequences, was used as negative control. At least four independent experiments were conducted for each gene.

### DNA isolation

Fungal genomic DNA was extracted from urediniospores as previously described [54]. Plant genomic DNA was isolated from healthy wheat leaves using a cetyltrimethylammonium bromide (CTAB) method [55]. To extract RNA and genomic DNA from the same leaf tissues, total RNA was isolated from *Pgt* infected leaf tissue at 12dpi using Trizol reagent (Invitrogen) according to the manufacturer’s instructions. Genomic DNA was then extracted from the interphase and phenol-chloroform layer. After removing any remaining aqueous solution overlying the interphase, 0.25 ml of BEB buffer (4 M guanidine thiocyanate, 50 mM sodium citrate, 1 M free base Tris) was added to 0.5 ml Trizol reagent used for RNA extraction. After incubating on a shaker for 10 minutes and centrifuging, the aqueous phase was recovered and DNA was precipitated by addition of 0.2 ml isopropanol and recovered by centrifugation.

### RT-qPCR analysis of transcript levels

To evaluate gene transcript levels in different developmental stages in *Pgt*, fresh urediniospores were collected from infected leaves at 12-14 dpi; germinated urediniospores were produced *in vitro* at room temperature; infected leaves were harvested at 5 dpi and haustoria were isolated from *Pgt* heavily infected leaves at 5 dpi. To evaluate the extent of gene silencing, the infected wheat leaves challenged with virus and rust fungi were harvested at 5 dpi for *Pgt* and *Pt* or 8 dpi for *Pst*. Total RNA was extracted using the Qiagen Plant RNeasy kit (Qiagen, Chatsworth, GA) according to the manufacturer’s instruction. RNA samples were treated with DNase I (Roche Diagnostics, Mannheim, Germany). The absence of genomic DNA contamination was subsequently confirmed by the null PCR amplification of RNA samples with primers designed for the *Pgt* Actin, *Pst* EF1 gene or the *Pt* succinate dehydrogenase gene [16, 26, 56]. RT-qPCR analysis was performed as described in Yin et al. [16, 19] to estimate candidate genes transcripts in different developmental stages and also to estimate levels of transcript after HIGS. To estimate transcript levels after HIGS, six biological replications were included for both the BSMV and the BSMV vector control constructs for each of the 15 genes. The transcript level of each putative silenced seedling leaf was compared with each of the BSMV control leaves, giving six estimates of silencing for each seedling. RT-qPCR was performed using a *Pgt*-actin, *Pst*-EF1 or *Pt* succinate dehydrogenase gene transcript to normalize the amount of cDNA in each of the samples from *Pgt*, *Pst*, or *Pt* respectively [16, 26, 56]*.*

To measure fungal biomass of infected leaves in HIGS assays, serial dilutions of total genomic DNA of *Pgt* urediniospores or uninfected wheat cultivar McNair 701 were used to make standard curves to correlate gene amplification with absolute DNA amounts. The quantification of DNA of the *Pgt*-actin and wheat-GAPDH genes in *Pgt* infected leaves was assessed by qPCR and this was used to determine the amount of fungal and plant DNA in the infected leaf samples. The relative amount of fungal biomass in different samples was presented as the ratio of fungal DNA to plant DNA in the samples.

### Statistical analysis

Data analyses were performed using the general linear models (GLM) procedures on JMP pro 11 statistical software (SAS Institute, Inc., Cary, NC). The LS Means statement was used to perform multiple comparisons. Differences at P ≤ 0.05 were considered statistically significant.

## Additional files

Additional file 1:
**Summary of the transcriptome sequencing of Pgt infected leaves and haustoria.** (DOCX 14 kb) Additional file 2:
**Summary of the selected haustoria expressed genes tested using VIGS assays.** (DOCX 26 kb) Additional file 3:
**Classifications of biological processes of the genes selected for silencing as determined by manual annotation and analysis with Blast 2G.** (DOCX 17 kb) Additional file 4:
**Primers used in this study.** (DOCX 28 kb)

## Abbreviations

BSMV: Barley stripe mosaic virus
HIGS: Host-induced gene silencing
*Pgt*: *Puccinia graminis* f. sp. *tritici*
*Pst*: *Puccinia striiformis* f. sp. *tritici*
*Pt*: *Puccinia triticina*
HR: Hypersensitive response
GHs: Glycoside hydrolases
FBA: Fructose-1, 6-bisphosphate aldolase
