# Supplementary material for: Identification of promising host-induced silencing targets among genes preferentially transcribed in haustoria of Puccinia
Source: BMC Genomics. 2015 Aug 5;16(1):579. doi: 10.1186/s12864-015-1791-y (PMC4524123; doi:10.1186/s12864-015-1791-y)
Supplement: Additional file 1: — Summary of the transcriptome sequencing of Pgt infected leaves and haustoria. (DOCX 14 kb) [file 12864_2015_1791_MOESM1_ESM.docx]

**Additional file 1. Summary of the transcriptome sequencing of *Pgt* infected leaves and haustoria.**

| **Library ID** | **Raw reads** | **Contamination (PhiX & Illumina adaptors)** | **Good quality reads** | **Reads with hits to wheat GI ver 12.0** | **% wheat** | **Reads hit to *Puccinia*** | **% *Puccinia*** |
| --- | --- | --- | --- | --- | --- | --- | --- |
| Pgt7A_InfW | 28,558,894 | 164,216 | 23,743,176 | 10,670,018 | 44.9% | 13,073,158 | 55.1% |
| Pgt7A_Haust | 16,000,237 | 1,262,528 | 13,336,892 | 2,673,800 | 20.0% | 10,663,092 | 80.0% |

InfW: infected wheat leaves; and Haust: purified haustoria.
