# Supplementary material for: Identification of promising host-induced silencing targets among genes preferentially transcribed in haustoria of Puccinia
Source: BMC Genomics. 2015 Aug 5;16(1):579. doi: 10.1186/s12864-015-1791-y (PMC4524123; doi:10.1186/s12864-015-1791-y)
Supplement: Additional file 2: — Summary of the selected haustoria expressed genes tested using VIGS assays. (DOCX 26 kb) [file 12864_2015_1791_MOESM2_ESM.docx]

**Additional file 2. Summary of the selected haustoria expressed genes tested using VIGS assays.**

| **Gene ID** | **Gene annotation** | **aa size** | **SP** | **TM** | **GOs** |
| --- | --- | --- | --- | --- | --- |
| PSTG_09151 | 5 -methylthioadenosine phosphorylase | 304 | N/A | N/A | F:transferase activity; P:heterocycle metabolic process; F:RNA binding; C:cytoplasm; C:nucleus |
| PSTG_16265 | aconitate hydratase | 778 | N/A | N/A | C:cytoplasm; P:biological_process; F:DNA binding; C:mitochondrion; P:mitochondrion organization; F:molecular_function; F:lyase activity; P:cellular respiration; P:cofactor metabolic process |
| PGTG_00315 | amino acid transporter | 540 | N/A | N/A | C:membrane; F:transporter activity; P:transport |
| PGTG_07026 | amino acid transporter | 532 | N/A | N/A | C:membrane; F:transporter activity; P:transport |
| PGTG_16914 | amino acid transporter | 557 | N/A | N/A | C:membrane; F:transporter activity; P:transport |
| PSTG_10170 | apc amino acid permease | 530 | N/A | N/A | C:membrane; F:transporter activity; P:transport |
| PSTG_14709 | ATP synthase F1 gamma | 307 | N/A | N/A | F:transporter activity; P:transport; P:heterocycle metabolic process; P:generation of precursor metabolites and energy; F:hydrolase activity; C:mitochondrial envelope; C:membrane |
| PSTG_00691 | chitin synthase | 895 | N/A | N/A | F:transferase activity; P:biological_process; C:membrane; C:cytoplasm |
| PGTG_04816 | extracellular invertase | 777 | N/A | Yes | P:biological_process; F:hydrolase activity |
| PSTG_11114 | extracellular invertase | 766 | Yes | N/A | P:biological_process; F:hydrolase activity |
| PGTG_14347 | FAD NAD -binding domain-containing protein | 479 | N/A | N/A | F:oxidoreductase activity; P:vitamin metabolic process; P:cofactor metabolic process; C:mitochondrial envelope; C:membrane; P:biological_process; P:heterocycle metabolic process; P:cellular aromatic compound metabolic process; |
| PGTG_15914 | family 18 glycoside hydrolase | 494 | Yes | N/A | F:hydrolase activity; F:molecular_function; P:biological_process |
| PGTG_01136 | fructose-bisphosphate class ii | 359 | N/A | N/A | P:cellular carbohydrate metabolic process; P:generation of precursor metabolites and energy; F:lyase activity; C:cytoplasm; F:molecular_function; C:mitochondrion; P:vitamin metabolic process; P:cofactor metabolic process; P:biological_process |
| PSTG_10983 | F-type h+-transporting atpase oligomycin sensitivity conferral protein | 214 | N/A | N/A | P:transport; P:heterocycle metabolic process; P:generation of precursor metabolites and energy; F:transporter activity; C:membrane |
| PGTG_17572 | glutamine amidotransferase | 353 | N/A | N/A | C:cytoplasm; F:peptidase activity; ; P:cofactor metabolic process |
| PGTG_10678 | glycerophosphoryl diester phosphodiesterase | 334 | N/A | N/A | P:cellular carbohydrate metabolic process; F:hydrolase activity; P:cellular lipid metabolic process |
| PGTG_01215 | glycoside hydrolase family 26 protein | 351 | Yes | N/A | F:hydrolase activity; P:cellular carbohydrate metabolic process |
| PGTG_00004 | glycoside hydrolase family 3 protein | 856 | Yes | N/A | P:biological_process; F:hydrolase activity |
| PGTG_03478 | glycoside hydrolase family 76 protein | 403 | Yes | N/A | F:hydrolase activity; P:cellular carbohydrate metabolic process |
| PGTG_04176 | glycosyltransferase family 31 protein | 679 | Yes | N/A | F:transferase activity |
| PGTG_18584 | HXT1p | 551 | N/A | Yes | P:transport; C:membrane; F:transporter activity |
| PGTG_21065 | [MFS monosaccharide transporter](http://blast.ncbi.nlm.nih.gov/Blast.cgi#alnHdr_630363935) | 583 | N/A | N/A | P:transport; C:membrane; F:transporter activity |
| PGTG_09355 | indoleamine 2,3-dioxyganese | 582 | N/A | N/A | F:molecular_function |
| PSTG_05275 | lysophospholipase plb1 | 615 | N/A | Yes | F:hydrolase activity; P:cellular lipid metabolic process |
| PGTG_11819 | mitochondrial intermediate peptidase | 791 | N/A | N/A | F:peptidase activity; P:biological_process |
| PGTG_00050 | mitochondrial processing peptidase beta subunit | 480 | N/A | N/A | F:molecular_function; P:transport; P:mitochondrion organization; F:peptidase activity; P:biological_process; C:mitochondrion |
| PGTG_17016 | oligopeptide transporter | 882 | N/A | N/A | P:transport |
| PGTG_15640 | phospholipase carboxylesterase | 268 | N/A | N/A | F:hydrolase activity |
| PGTG_14350 | ABC transporter-like protein | 339 | Yes | N/A | P:transport |
| PGTG_16572 | siderochrome-iron transporter | 673 | N/A | N/A | P:transport; C:membrane |
| PGTG_01304 | thiazole biosynthetic enzyme | 336 | N/A | N/A | P:vitamin metabolic process; P:cellular aromatic compound metabolic process; P:heterocycle metabolic process; C:cytoplasm; F:molecular_function; C:nucleus |
| PGTG_07223 | transcription factor ste12 | 848 | N/A | N/A | F:DNA binding; F:transcription regulator activity; F:molecular_function; P:RNA metabolic process; ; C:nucleus |
| PGTG_04902 | translation initiation factor eif-5 | 405 | N/A | N/A | P:RNA metabolic process; F:molecular_function; F:protein binding; C:ribosome; P:translation |
| PSTG_06514 | triosephosphate isomerase | 293 | N/A | N/A | F:isomerase activity; P:cellular carbohydrate metabolic process; P:generation of precursor metabolites and energy; P:biological_process; P:cellular lipid metabolic process |
| PGTG_11658 | tryptophan 2-monooxygenase | 588 | N/A | N/A | F:oxidoreductase activity; P:biological_process |
| PGTG_00860 | UDP-glucose:sterol glucosyltransferase | 513 | N/A | N/A | P:biological_process; P:cellular lipid metabolic process; F:transferase activity |
| PGTG_10303 | voltage-gated potassium channel beta-2 subunit | 385 | N/A | N/A | F:ion channel activity |
| PSTG_16715 | osmotin thaumatin-like protein | 183 | Yes | Yes | - |
| PGTG_10642 | secreted protein | 150 | Yes | N/A | - |
| PGTG_08617 | secreted protein | 161 | Yes | N/A | - |
| PGTG_15486 | secreted protein | 191 | Yes | N/A | - |
| PGTG_02151 | secreted protein | 192 | Yes | N/A | - |
| PGTG_10731 | hypothetical protein | 280 | N/A | Yes | - |
| PGTG_00120 | hypothetical protein | 201 | Yes | N/A | - |
| PGTG_10923 | hypothetical protein | 580 | N/A | N/A | - |
| PGTG_08701 | hypothetical protein | 343 | N/A | N/A | - |
| PGTG_07754 | hypothetical protein | 392 | N/A | N/A | - |
| PGTG_04242 | hypothetical protein | 283 | N/A | N/A | - |
| PGTG_10046 | hypothetical protein | 257 | N/A | N/A | - |
| PGTG_15003 | hypothetical protein | 299 | N/A | N/A | - |
| PGTG_08739 | hypothetical protein | 1155 | N/A | N/A | - |
| PGTG_02947 | hypothetical protein | 258 | N/A | N/A | - |
| PGTG_03050 | hypothetical protein | 303 | N/A | N/A | - |
| PGTG_17724 | hypothetical protein | 117 | N/A | Yes | - |
| PGTG_17153 | hypothetical protein | 191 | N/A | Yes | - |
| PGTG_17788 | hypothetical protein | 462 | N/A | Yes | - |
| PGTG_00194 | hypothetical protein | 402 | Yes | N/A | - |
| PGTG_03101 | hypothetical protein | 251 | Yes | N/A | - |
| PGTG_03590 | hypothetical protein | 128 | Yes | N/A | - |
| PGTG_04476 | hypothetical protein | 737 | Yes | N/A | - |
| PGTG_07422 | hypothetical protein | 721 | N/A | N/A | - |
| PGTG_07423 | hypothetical protein | 475 | N/A | N/A | - |
| PGTG_08644 | hypothetical protein | 255 | Yes | N/A | - |
| PGTG_08762 | hypothetical protein | 171 | Yes | N/A | - |
| PGTG_09204 | hypothetical protein | 114 | Yes | N/A | - |
| PGTG_10261 | hypothetical protein | 1186 | N/A | Yes | - |
| PGTG_10751 | hypothetical protein | 235 | Yes | N/A | - |
| PGTG_11199 | hypothetical protein | 116 | Yes | N/A | - |
| PGTG_12371 | hypothetical protein | 384 | N/A | N/A | - |
| PGTG_13410 | hypothetical protein | 134 | Yes | N/A | - |
| PGTG_14615 | hypothetical protein | 245 | N/A | N/A | - |
| PGTG_15507 | hypothetical protein | 111 | Yes | N/A | - |
| PGTG_15927 | hypothetical protein | 132 | Yes | N/A | - |
| PGTG_16158 | hypothetical protein | 137 | Yes | N/A | - |
| PGTG_16225 | hypothetical protein | 310 | Yes | N/A | - |
| PGTG_16227 | hypothetical protein | 637 | N/A | N/A | - |
| PGTG_17020 | hypothetical protein | 353 | Yes | N/A | - |
| PGTG_06692 | hypothetical protein | 340 | N/A | Yes | - |
| PGTG_09404 | hypothetical protein | 501 | N/A | N/A | - |
| PGTG_12202 | hypothetical protein | 138 | N/A | N/A | - |
| PGTG_10406 | hypothetical protein | 425 | N/A | Yes | - |
| PGTG_11120 | hypothetical protein | 524 | Yes | N/A | - |
| PGTG_12890 | hypothetical protein | 506 | N/A | Yes | - |
| PGTG_03216 | hypothetical protein | 217 | N/A | N/A | - |
| PGTG_00656 | hypothetical protein | 640 | N/A | N/A | - |
| PGTG_07000 | hypothetical protein | 202 | Yes | N/A | - |

PGTG: *Pgt* gene; PSTG: *Pst* gene; Sp: signal peptide; TM: transmembrane domain
