# Supplementary material for: Identification of promising host-induced silencing targets among genes preferentially transcribed in haustoria of Puccinia
Source: BMC Genomics. 2015 Aug 5;16(1):579. doi: 10.1186/s12864-015-1791-y (PMC4524123; doi:10.1186/s12864-015-1791-y)
Supplement: Additional file 3: — Classifications of biological processes of the genes selected for silencing as determined by manual annotation and analysis with Blast 2G. (DOCX 17 kb) [file 12864_2015_1791_MOESM3_ESM.docx]

**Additional file 3. Biological process classification of the genes selected for silencing (analyzed by Blast 2G).**
