# Supplementary material for: Identification of promising host-induced silencing targets among genes preferentially transcribed in haustoria of Puccinia
Source: BMC Genomics. 2015 Aug 5;16(1):579. doi: 10.1186/s12864-015-1791-y (PMC4524123; doi:10.1186/s12864-015-1791-y)
Supplement: Additional file 4: — Primers used in this study. (DOCX 28 kb) [file 12864_2015_1791_MOESM4_ESM.docx]

**Additional file 4. Primers used in this study.**

| **Primer** | **Sequence 5' → 3'** | **Purpose** |
| --- | --- | --- |
| PGTG_00004F | ATAAGAATGCGGCCGCTAAACTATTAGCGATGCCGGCGACAATC | gene silencing |
| PGTG_00004R | CCTTAATTAAGGTGCAAACCTGCGCGGTTTTG | gene silencing |
| PGTG_00050F | ATAAGAATGCGGCCGCTAAACTATCACTCTCAAACAGCTACCGTTG | gene silencing |
| PGTG_00050R | CCTTAATTAAGGGTCCGAAATGATCTCGACTACT | gene silencing |
| PGTG_00120F | ATAAGAATGCGGCCGCTAAACTATCTTCCTCCCACAAGACTGGT | gene silencing |
| PGTG_00120R | CCTTAATTAAGGGGTTCGGAGAAGATGCCAAA | gene silencing |
| PGTG_00194F | ATAAGAATGCGGCCGCTAAACTATACAAAAAAACCCGAACCAGAG | gene silencing |
| PGTG_00194R | CCTTAATTAAGGCAGAATTCTGAGCGGACGTGAA | gene silencing |
| PGTG_00315F | ATAAGAATGCGGCCGCTAAACTATAAAAGGCTTGTCATCGGTAATC | gene silencing |
| PGTG_00315R | CCTTAATTAAGGTGCTTTTCCGTGACTAGCGG | gene silencing |
| PGTG_00656F | ATAAGAATGCGGCCGCTAAACTATGTTCCAAAAGCTATTGCATACC | gene silencing |
| PGTG_00656R | CCTTAATTAAGGTGTACACGGCAAGCGTCTTT | gene silencing |
| PGTG_00860F | ATAAGAATGCGGCCGCTAAACTATCATGAATGCCATCATAGGTGCG | gene silencing |
| PGTG_00860R | CCTTAATTAAGGAGCGGCATCTATCTGGTCGAAC | gene silencing |
| PGTG_01136F | ATAAGAATGCGGCCGCTAAACTATGGGACGTTTATTCTGCTTTCAG | gene silencing |
| PGTG_01136R | CCTTAATTAAGGTTTCCAAGGAGTTCGGGTTGC | gene silencing |
| PGTG_01215F | ATAAGAATGCGGCCGCTAAACTATCCCTTACGGCTAAAATTGATGG | gene silencing |
| PGTG_01215R | CCTTAATTAAGGGCATTACCGGGGTATTCGTG | gene silencing |
| PGTG_01304F | ATAAGAATGCGGCCGCTAAACTATAATCCAACCAGGCTGCCCCA | gene silencing |
| PGTG_01304R | CCTTAATTAAGGCACGACAATCCCGCCGAACC | gene silencing |
| PGTG_02151F | ATAAGAATGCGGCCGCTAAACTATATGTTTTTCCAAACCCGCTC | gene silencing |
| PGTG_02151R | CCTTAATTAAGGTTCCACTGAGTTCAACCCGC | gene silencing |
| PGTG_02947F | ATAAGAATGCGGCCGCTAAACTATAACTACCCAGGACGCTCAGC | gene silencing |
| PGTG_02947R | CCTTAATTAAGG AACTGAAGCCCTGTATCCTAG | gene silencing |
| PGTG_03050F | ATAAGAATGCGGCCGCTAAACTATTTCAGCTTCACCCACGGGTT | gene silencing |
| PGTG_03050R | CCTTAATTAAGGGGATTCGAACCATTCCTTGTTG | gene silencing |
| PGTG_03101F | ATAAGAATGCGGCCGCTAAACTATCAGCAAGGAATGCCACAGAG | gene silencing |
| PGTG_03101R | CCTTAATTAAGGGCCCAGAATCTGGTTGAAGC | gene silencing |
| PGTG_03216F | ATAAGAATGCGGCCGCTAAACTATTGACACTCGATACCCCGTCCGT | gene silencing |
| PGTG_03216R | CCTTAATTAAGGTATCGGTCGTCGTAGCGGTCTC | gene silencing |
| PGTG_03478F | ATAAGAATGCGGCCGCTAAACTATCGAATTTTTAGGACCACAGGCC | gene silencing |
| PGTG_03478R | CCTTAATTAAGGGTTGAATGCCTTGTACCTTCCA | gene silencing |
| PGTG_03590F | ATAAGAATGCGGCCGCTAAACTATTGTTTACGGATCAGCCCCAGTT | gene silencing |
| PGTG_03590R | CCTTAATTAAGGAGGTGTTGGTGTCCTGGTTGAA | gene silencing |
| PGTG_04176F | ATAAGAATGCGGCCGCTAAACTATTGCCGATAGGTTCGAGAGACGGT | gene silencing |
| PGTG_04176R | CCTTAATTAAGGTCACGACTTGTTGAACATCCCGA | gene silencing |
| PGTG_04242F | ATAAGAATGCGGCCGCTAAACTATTGTCAGCCCACTATCTTGGT | gene silencing |
| PGTG_04242R | CCTTAATTAAGGCTATTTACTACCAGCTATGAAGG | gene silencing |
| PGTG_04476F | ATAAGAATGCGGCCGCTAAACTATAACCCCGGTAACTCCCCC | gene silencing |
| PGTG_04476R | CCTTAATTAAGGTGGCTGCTGCTGCTGCTG | gene silencing |
| PGTG_04816F | ATAAGAATGCGGCCGCTAAACTAT CCCGTCACCAAACGAGTGGAAA | gene silencing |
| PGTG_04816R | CCTTAATTAAGGAATGGACGGAATGAGGCTGGAA | gene silencing |
| PGTG_04902F | ATAAGAATGCGGCCGCTAAACTATAGGCGACCTCGACACTTAAGGA | gene silencing |
| PGTG_04902R | CCTTAATTAAGGTCAGCCAGACTCAGTCCATTCT | gene silencing |
| PGTG_06692F | ATAAGAATGCGGCCGCTAAACTATTACGCCCATATACCCTGGTG | gene silencing |
| PGTG_06692R | CCTTAATTAAGGGCTCCAACAATAGCTCGGCA | gene silencing |
| PGTG_07000F | ATAAGAATGCGGCCGCTAAACTATTCATCAACATCGACCGTCCA | gene silencing |
| PGTG_07000R | CCTTAATTAAGGAGCTTGATGTACGCTCCAAG | gene silencing |
| PGTG_07026F | ATAAGAATGCGGCCGCTAAACTATATTCGTTTGCGATTTGCGTGGC | gene silencing |
| PGTG_07026R | CCTTAATTAAGGAGAAAGCTGTAGGCTGCCTGGA | gene silencing |
| PGTG_07223F | ATAAGAATGCGGCCGCTAAACTATTCCAATCACACCCAGCCAGGTT | gene silencing |
| PGTG_07223R | CCTTAATTAAGGTAATGAGGTCCTCCTGGTCCGT | gene silencing |
| PGTG_07422F | ATAAGAATGCGGCCGCTAAACTATAGCGGCAAGCACCCTTTCAC | gene silencing |
| PGTG_07422R | CCTTAATTAAGGCGTCGTCTGGGCATCCTCAT | gene silencing |
| PGTG_07423F | ATAAGAATGCGGCCGCTAAACTATAGGGTTGTTTGAACTCAGGCGC | gene silencing |
| PGTG_07423R | CCTTAATTAAGGGGAAGGTCGGACCCATATACTT | gene silencing |
| PGTG_07754F | ATAAGAATGCGGCCGCTAAACTATAGAACTCTTCCCAGTGCCAA | gene silencing |
| PGTG_07754R | CCTTAATTAAGGATCCCGTGTGCCAAGTTAGA | gene silencing |
| PGTG_08617F | ATAAGAATGCGGCCGCTAAACTATGCTTTTGAATGCACTGGTGGTT | gene silencing |
| PGTG_08617R | CCTTAATTAAGGGGAGCTGTTGCATGTGAAATGG | gene silencing |
| PGTG_08644F | ATAAGAATGCGGCCGCTAAACTATCCCAGAAATGTTAACCCCAGAA | gene silencing |
| PGTG_08644R | CCTTAATTAAGGCTCTCGGAACTGCTCAACAAGT | gene silencing |
| PGTG_08701F | ATAAGAATGCGGCCGCTAAACTATGGAGTTAAAGACGACTTACCTT | gene silencing |
| PGTG_08701R | CCTTAATTAAGGAGATCTAGCTTGACGTGCACTC | gene silencing |
| PGTG_08739F | ATAAGAATGCGGCCGCTAAACTATTTCCGGCTCGCCTCTGAGCTCT | gene silencing |
| PGTG_08739R | CCTTAATTAAGGACTGGAAGAGAGCTCAAGGGAG | gene silencing |
| PGTG_08762F | ATAAGAATGCGGCCGCTAAACTATAGGAGACGATTCAGGAAGCAAC | gene silencing |
| PGTG_08762R | CCTTAATTAAGGGTAAGGGCAAGGGTTCCAATGT | gene silencing |
| PGTG_09204F | ATAAGAATGCGGCCGCTAAACTATTCGTCTGCGGGCTCATCCTT | gene silencing |
| PGTG_09204R | CCTTAATTAAGGCGGTGCTGACTACACACCAA | gene silencing |
| PGTG_09355F | ATAAGAATGCGGCCGCTAAACTATAACAACCTTCACACGATCGGAC | gene silencing |
| PGTG_09355R | CCTTAATTAAGGGGACGTCACTCAGAACCTTTGA | gene silencing |
| PGTG_09404F | ATAAGAATGCGGCCGCTAAACTATGACTTCATCACATATCCTTTCG | gene silencing |
| PGTG_09404R | CCTTAATTAAGGGTCATGAGCGTTTTTGGTCTG | gene silencing |
| PGTG_10046F | ATAAGAATGCGGCCGCTAAACTATAAAGCACTCAGTGATAATCTACCC | gene silencing |
| PGTG_10046R | CCTTAATTAAGGTCGCTTTCAATCTCAAATCCATCC | gene silencing |
| PGTG_10261F | ATAAGAATGCGGCCGCTAAACTATTGCGCACGTCCCATTACAAC | gene silencing |
| PGTG_10261R | CCTTAATTAAGGGAGACGATCGAGAGTGAATG | gene silencing |
| PGTG_10303F | ATAAGAATGCGGCCGCTAAACTATTTGGGTCGGCTCCAGCTCAG | gene silencing |
| PGTG_10303R | CCTTAATTAAGGCAGTAGAAAGCCAAGCGCTGAT | gene silencing |
| PGTG_10406F | ATAAGAATGCGGCCGCTAAACTATGATCTACAATCTGCCTACGAGC | gene silencing |
| PGTG_10406R | CCTTAATTAAGGTCATCCTTGTAACTAGTCGCCG | gene silencing |
| PGTG_10642F | ATAAGAATGCGGCCGCTAAACTATCCTCACCAACGGGCTTTCTAAG | gene silencing |
| PGTG_10642R | CCTTAATTAAGGTAAAGTAATGCCGGAGTTCGGG | gene silencing |
| PGTG_10678F | ATAAGAATGCGGCCGCTAAACTATTTTCTCCCGTGAACAAGGCGAA | gene silencing |
| PGTG_10678R | CCTTAATTAAGGGGCTTCAAATGAAGACAGCGTG | gene silencing |
| PGTG_10731F | ATAAGAATGCGGCCGCTAAACTATGCATTTCTGATATTTTCGGCCG | gene silencing |
| PGTG_10731R | CCTTAATTAAGGATCCCTCTTTGCCAATCGCC | gene silencing |
| PGTG_10751F | ATAAGAATGCGGCCGCTAAACTATTTACAAGCGACGTAGAGGGC | gene silencing |
| PGTG_10751R | CCTTAATTAAGGTGCCAACTTCCAGCGAAACG | gene silencing |
| PGTG_10923F | ATAAGAATGCGGCCGCTAAACTATGACTGGAAGTATTCCACAATTC | gene silencing |
| PGTG_10923R | CCTTAATTAAGGCACTCTTCTTTGAAGCCCTATC | gene silencing |
| PGTG_11120F | ATAAGAATGCGGCCGCTAAACTATTAGGCAGCGAAGTTCTTACACC | gene silencing |
| PGTG_11120R | CCTTAATTAAGGCGAAGAATCCAGCGGCGAAGTT | gene silencing |
| PGTG_11199F | ATAAGAATGCGGCCGCTAAACTATGCAGAGCTTCAACTTATTCATCG | gene silencing |
| PGTG_11199R | CCTTAATTAAGGTCAGCCTTATGCTCATCAGCAG | gene silencing |
| PGTG_11819F | ATAAGAATGCGGCCGCTAAACTATAGCAAGGACCCTATCCATCCCT | gene silencing |
| PGTG_11819R | CCTTAATTAAGGATCGAAGAGGTCGCAGTAGATG | gene silencing |
| PGTG_12202F | ATAAGAATGCGGCCGCTAAACTATATGAGGCTATTTTACGGCAGCG | gene silencing |
| PGTG_12202R | CCTTAATTAAGGTTTCTGTTCCGCAAGTCTTTCG | gene silencing |
| PGTG_12371F | ATAAGAATGCGGCCGCTAAACTATCCTATCTCGATGGCAGACACGA | gene silencing |
| PGTG_12371R | CCTTAATTAAGGAGGGCTATCAACAGTGGTGATC | gene silencing |
| PGTG_12890F | ATAAGAATGCGGCCGCTAAACTATATGCATCAGGATCAGGGGAG | gene silencing |
| PGTG_12890R | CCTTAATTAAGGACTGGGGTTTGTGGAACTGA | gene silencing |
| PGTG_13410F | ATAAGAATGCGGCCGCTAAACTATTTTGCCCAAGGTTGACCGAATG | gene silencing |
| PGTG_13410R | CCTTAATTAAGGAGCAGGATCATCTGCCTTTCCA | gene silencing |
| PGTG_14347F | ATAAGAATGCGGCCGCTAAACTATCCACTCGATGGTCCCCTTTTAC | gene silencing |
| PGTG_14347R | CCTTAATTAAGGAAACCTTGTAACCTTGTCGCTC | gene silencing |
| PGTG_14350F | ATAAGAATGCGGCCGCTAAACTATAACTTAAGAGACTCCGTCAACG | gene silencing |
| PGTG_14350R | CCTTAATTAAGGCGTGTCCTGGATGTATTTGACA | gene silencing |
| PGTG_14615F | ATAAGAATGCGGCCGCTAAACTATCGACGCTCGGACAACTTATCGA | gene silencing |
| PGTG_14615R | CCTTAATTAAGGCCAGGCCCGACAAGAGTAGA | gene silencing |
| PGTG_15003F | ATAAGAATGCGGCCGCTAAACTATGGGCTTCTCCATGGTCGATT | gene silencing |
| PGTG_15003R | CCTTAATTAAGGGATCGAGCCAGGCCATGCAG | gene silencing |
| PGTG_15486F | ATAAGAATGCGGCCGCTAAACTATATCACAAACTTCGTCAGCTCGA | gene silencing |
| PGTG_15486R | CCTTAATTAAGGATGGCGTCATCAAGTCCACC | gene silencing |
| PGTG_15507F | ATAAGAATGCGGCCGCTAAACTATGGCTACACCGATATGCTTTGTT | gene silencing |
| PGTG_15507R | CCTTAATTAAGGGCAATTGTTCCGGTAACACACC | gene silencing |
| PGTG_15640F | ATAAGAATGCGGCCGCTAAACTATGTCTTCTTGTGGACTCGAAG | gene silencing |
| PGTG_15640R | CCTTAATTAAGGGTTTCGACCAGAATTCAAGG | gene silencing |
| PGTG_15914F | ATAAGAATGCGGCCGCTAAACTATATCCTGGACCTTGACTAAGCCC | gene silencing |
| PGTG_15914R | CCTTAATTAAGGTAGCAAGGAATGGCTCTCCGGA | gene silencing |
| PGTG_15927F | ATAAGAATGCGGCCGCTAAACTATGATCACTACGTCTGTCATGTCG | gene silencing |
| PGTG_15927R | CCTTAATTAAGGATGGTCTCTTCCCAGATCCAAC | gene silencing |
| PGTG_16158F | ATAAGAATGCGGCCGCTAAACTATTTGATAGGGGTTTACAAGGCCC | gene silencing |
| PGTG_16158R | CCTTAATTAAGGAGACCAACTTCATCCAGAAGTG | gene silencing |
| PGTG_16225F | ATAAGAATGCGGCCGCTAAACTATAAGAATGGCAATGCGGCTCCAT | gene silencing |
| PGTG_16225R | CCTTAATTAAGGCAAGCTCCGAACTTGAGCGTAT | gene silencing |
| PGTG_16227F | ATAAGAATGCGGCCGCTAAACTATAGACACCCTCAGCCTCCTAC | gene silencing |
| PGTG_16227R | CCTTAATTAAGGTCCGCCCATCCGTAGATGTG | gene silencing |
| PGTG_16572F | ATAAGAATGCGGCCGCTAAACTATCAAGACGAGGCAGAAAAGGCTA | gene silencing |
| PGTG_16572R | CCTTAATTAAGGCATGGAGAGGTAATTGTCGAGG | gene silencing |
| PGTG_16914F | ATAAGAATGCGGCCGCTAAACTATCATGACAGTAGCTTTGGGAGAG | gene silencing |
| PGTG_16914R | CCTTAATTAAGGAATCCTGTCGTGAGTGGGTGT | gene silencing |
| PGTG_17016F | ATAAGAATGCGGCCGCTAAACTATGCTGCCCGACGTATTCTTTCA | gene silencing |
| PGTG_17016R | CCTTAATTAAGGTATGCCACTGATAGCCGCGA | gene silencing |
| PGTG_17020F | ATAAGAATGCGGCCGCTAAACTATGCTTTATCATGCCAAGTCCTC | gene silencing |
| PGTG_17020R | CCTTAATTAAGGTTGGTAGCAGCGTCATTTGC | gene silencing |
| PGTG_17153F | ATAAGAATGCGGCCGCTAAACTATTCTGACGCTGTACGCAATAG | gene silencing |
| PGTG_17153R | CCTTAATTAAGGATGCGAAATACAGCTGGACC | gene silencing |
| PGTG_17572F | ATAAGAATGCGGCCGCTAAACTATTAGCCGATCCGAAATCGGTC | gene silencing |
| PGTG_17572R | CCTTAATTAAGGGTCGAGAGCACTAGCAGCTT | gene silencing |
| PGTG_17724F | ATAAGAATGCGGCCGCTAAACTATGTCCGTGAACCTCAAGCTCAAT | gene silencing |
| PGTG_17724R | CCTTAATTAAGGTCAAAGGCAGTGGAAGCGGCAA | gene silencing |
| PGTG_17788F | ATAAGAATGCGGCCGCTAAACTATCTTCAGAGCAATTCTTGGTCTG | gene silencing |
| PGTG_17788R | CCTTAATTAAGGTGTACGACCCAAGCATGGTT | gene silencing |
| PGTG_18584F | ATAAGAATGCGGCCGCTAAACTATATTTGCAGCAACGACTGACGCT | gene silencing |
| PGTG_18584R | CCTTAATTAAGGGCTCCAGCAATCAACATCGTCC | gene silencing |
| Pgt-IaaMF | ATAAGAATGCGGCCGCTAAACTATCAAGTCTTGGAGCATTCACTCTGG | gene silencing |
| Pgt-IaaMR | CCTTAATTAAGGGACATTCATGGAAGTCCTCAACGC | gene silencing |
| PGTG_21065F | ATAAGAATGCGGCCGCTAAACTATGATGATGACTATCTGTCATCTCAG | gene silencing |
| PGTG_21065R | CCTTAATTAAGG GGGGAAAATTTCCGAAGGCA | gene silencing |
| PSTG_00691F | CCTTAATTAAGGTCGGGAAGCAATTCTCGCAT | gene silencing |
| PSTG_00691R | ATAAGAATGCGGCCGCTAAACTATAATGTGGGCAACGGTCTCTT | gene silencing |
| PSTG_03360F | CCTTAATTAAGGATGGGTGGTTTACTCGAACTCG | gene silencing |
| PSTG_03360R | ATAAGAATGCGGCCGCTAAACTATGAGCTTCTTTGCACAATGGTCTG | gene silencing |
| PSTG_04507F | CCTTAATTAAGGGCAATCCACTAACTGCCAATCAC | gene silencing |
| PSTG_04507R | ATAAGAATGCGGCCGCTAAACTATCATGGTGCGTAGCGATGCAAATA | gene silencing |
| PSTG_04871F | CCTTAATTAAGGGAATACCGGAAATATGCACCCGAC | gene silencing |
| PSTG_04871R | ATAAGAATGCGGCCGCTAAACTATCTGTCAAAAGTTTGGTGGAAACGC | gene silencing |
| PSTG_05275F | CCTTAATTAAGGCAGCCCTTACCACATCACCT | gene silencing |
| PSTG_05275R | ATAAGAATGCGGCCGCTAAACTATCAAGCTCCCAAAAGCCAACT | gene silencing |
| PSTG_06514F | CCTTAATTAAGGCATTCAAGTCGGTGCCCAAA | gene silencing |
| PSTG_06514R | ATAAGAATGCGGCCGCTAAACTATTGACACTACCGCCGTAAATGA | gene silencing |
| PSTG_09151F | CCTTAATTAAGGGATTCGAGCCCAACGATCAGA | gene silencing |
| PSTG_09151R | ATAAGAATGCGGCCGCTAAACTATACCGCATTGTGAACCTCATC | gene silencing |
| PSTG_10170F | CCTTAATTAAGGGGGCACGGGAATATTTGGTT | gene silencing |
| PSTG_10170R | ATAAGAATGCGGCCGCTAAACTATGCGTATGAGGAGTCTAGGGC | gene silencing |
| PSTG_10983F | CCTTAATTAAGGGTCGTCTGGATCAGTCTACCG | gene silencing |
| PSTG_10983R | ATAAGAATGCGGCCGCTAAACTATCATGTCAGATAAGCCTTTCTTGCG | gene silencing |
| PSTG_11114F | CCTTAATTAAGGTACGGCCGAGTACAACCAAA | gene silencing |
| PSTG_11114R | ATAAGAATGCGGCCGCTAAACTATAATGTAAAAGCTCCTTGCGCC | gene silencing |
| PSTG_11830F | CCTTAATTAAGG CACTGAGCCTGGCGATAACACTT | gene silencing |
| PSTG_11830R | ATAAGAATGCGGCCGCTAAACTAT CTCAGATCCCAATATCCTGAAGC | gene silencing |
| PSTG_14709F | CCTTAATTAAGGCATTCCAGCGTTTCTAAGCGT | gene silencing |
| PSTG_14709R | ATAAGAATGCGGCCGCTAAACTATTCAACTTCGTAAGCGGCAAAC | gene silencing |
| PSTG_16265F | CCTTAATTAAGGAGCCATGCATCCAAGATCGG | gene silencing |
| PSTG_16265R | ATAAGAATGCGGCCGCTAAACTATCATCTGGGCTGTGGCATCTT | gene silencing |
| PSTG_16715F | CCTTAATTAAGGCACCGAATGGACAACCCGAT | gene silencing |
| PSTG_16715R | ATAAGAATGCGGCCGCTAAACTATTCGAAAGTAGAACCAACGGGG | gene silencing |
| PGTG_18333F | ATAAGAATGCGGCCGCTAAACTATCGGCCTCTATCTTGGTGAAGTT | gene silencing |
| PGTG_18333R | CCTTAATTAAGGATCCGGTTTCGACCATTCCCAT | gene silencing |
| PGTG_07534F | ATAAGAATGCGGCCGCTAAACTATGCCAAGCATTTCGTTGCACTGT | gene silencing |
| PGTG_07534R | CCTTAATTAAGGATCGGCCCTGTTTGATACGTCA | gene silencing |
| PGTG_000384F | ATAAGAATGCGGCCGCTAAACTATATCTTGACCCCAACACAGAGGT | gene silencing |
| PGTG_000384R | CCTTAATTAAGGACCACAACTCGGTCCCAGGATT | gene silencing |
| PGTG_04956F | ATAAGAATGCGGCCGCTAAACTATCCAGTCAAACTTGGAGTCAACG | gene silencing |
| PGTG_04956R | CCTTAATTAAGGCGCCTGCTTGGTGGTAAATACA | gene silencing |
| PGTG_17029F | ATAAGAATGCGGCCGCTAAACTATTCGACGAATGTGTTGGTCCCAA | gene silencing |
| PGTG_17029R | CCTTAATTAAGGCTTTCTTCATCAAGAAGCCGGC | gene silencing |
| PGTG_04139F | ATAAGAATGCGGCCGCTAAACTATTGAACACAGGCGAAACTCATGC | gene silencing |
| PGTG_04139R | CCTTAATTAAGGTTGCTTTTGGCATAACCGAGGG | gene silencing |
| PGTG_14903F | ATAAGAATGCGGCCGCTAAACTATGTCCAAGGCGCTGAAGAATCTC | gene silencing |
| PGTG_14903R | CCTTAATTAAGGCGACACGATTCCGTACTTCTTG | gene silencing |
| PGTG_16473F | ATAAGAATGCGGCCGCTAAACTATCGAAATTCCTGCCAGTCAAGTC | gene silencing |
| PGTG_16473R | CCTTAATTAAGGGGGGATAACACTAAATCGGCTG | gene silencing |
| PGTG_05736F | ATAAGAATGCGGCCGCTAAACTATAACAGGGGCAGCGGTAGTTCAT | gene silencing |
| PGTG_05736R | CCTTAATTAAGGGAAATCAGCCCACCAAGGCTTG | gene silencing |
| PGTG_01136RTF | CCGATCATTGTGCCAAGAAAC | RT-qPCR |
| PGTG_01136RTR | CGAGCATGTGAGACGAGAATAA | RT-qPCR |
| PGTG_01215RTF | GGTGCCCGAATGTATCAACTA | RT-qPCR |
| PGTG_01215RTR | GGTAGTAGTCAAATCCGACGATG | RT-qPCR |
| PGTG_01304RTF | CGAAAGCCTGCCGACAAATTCT | RT-qPCR |
| PGTG_01304RTR | GGATAAGACGGTCGAGGTAAAC | RT-qPCR |
| PGTG_03478RTF | ACGCCTCGCAGATGTTAAGAC | RT-qPCR |
| PGTG_03478RTR | CAGCCATAGGCCATAGTTGTAG | RT-qPCR |
| PGTG_03590RTF | GCCTGATCTATCACGAGACTTATG | RT-qPCR |
| PGTG_03590RTR | GGCTGATCCGTAAACATATACCA | RT-qPCR |
| PGTG_10731RTF | ATAACATGCCACCAGAGTATCG | RT-qPCR |
| PGTG_10731RTR | CGGATCAGACAAACTGACTCTT | RT-qPCR |
| PGTG_12890RTF | CGCAATCCAACAACACGTTAG | RT-qPCR |
| PGTG_12890RTR | CCCGATCCGTTTCTCCATTAT | RT-qPCR |
| PGTG_14350RTF | CTACAAACTCATCGGCACCTA | RT-qPCR |
| PGTG_14350RTR | GGACGACTGATTCCCATCTTT | RT-qPCR |
| PGTG_16914RTF | GACTGGTCCACCTCAGATTTTC | RT-qPCR |
| PGTG_16914RTR | ACTGCCTTTTCTCTCTCCAGTG | RT-qPCR |
| Pgt-IaaMRTF | GGGCAACAAGAATGGGAAGA | RT-qPCR |
| Pgt-IaaMRTR | CCACTAAGCGGCAGATGTAAG | RT-qPCR |
| PSTG_03360RTF | ACTGACACTCAATGGGCTTACA | RT-qPCR |
| PSTG_03360RTR | CAAACTCGTGGGTCGTAGTATT | RT-qPCR |
| PSTG_04507RTF | GCGGTTATGGTCCTGTCTATTC | RT-qPCR |
| PSTG_04507RTR | GTCGACCATCCTTCATCTTTGC | RT-qPCR |
